# Highly Porous Carbon Materials Derived from Silicon Oxycarbides and Effect of the Pyrolysis Temperature on Their Electrochemical Response

**DOI:** 10.3390/ijms241813868

**Published:** 2023-09-08

**Authors:** Jose Merida, Maria T. Colomer, Fausto Rubio, M. Alejandra Mazo

**Affiliations:** 1 Departamento de Ingeniería Química, Universidad Autónoma de Madrid, C/Tomás y Valiente 7, 28049 Madrid, Spain; josseantoniomeridaa@gmail.com; 2 Instituto de Cerámica y Vidrio, Consejo Superior de Investigaciones Científicas, C/Kelsen 5, 28049 Madrid, Spain; tcolomer@icv.csic.es (M.T.C.); frubio@icv.csic.es (F.R.)

**Keywords:** hierarchical porous materials, micro-meso-macroporous materials, silicon oxycarbide carbon derived materials, chlorine etching, Raman parameters, supercapacitor applications

## Abstract

The design of a material porous microstructure with interconnected micro-meso-macropores is a key issue for the successful development of carbon-derived materials for supercapacitor applications. Another important issue is the nature of these carbon materials. For those reasons, in this study, novel hierarchical micro-meso-macroporous silicon oxycarbide-derived carbon (SiOC-DC) was obtained via chlorine etching of carbon-enriched SiOC prepared via pyrolysis (1100–1400 °C) of sol-gel triethoxysilane/dimethyldiphenysiloxane hybrids. In addition, and for the first time, non-conventional Raman parameters combined with the analysis of their microstructural characteristics were considered to establish their relationships with their electrochemical response. The sample pyrolyzed at 1100 °C showed planar and less-defective carbon domains together with the largest specific surface area (SSA) and the highest volume of micro-meso-macropores, which upgraded their electrochemical response. This sample has the highest specific capacitance (C_s_ = 101 Fg^−1^ (0.2 Ag^−1^)), energy (E_d_ = 12–7 Wh^−1^ kg^−1^), and power densities (P_d_ = 0.32–35 kw kg^−1^), showing a good capacitance retention ratio up to 98% after 10,000 charge–discharge cycles at 0.5 Ag^−1^. At a pyrolysis temperature ≥ 1200 °C, the carbon domains were highly ordered and tortuous with a high degree of interconnection. However, SSA and pore volumes (micro-meso-macropores) were significantly reduced and downgraded the C_s_, E_d_, and P_d_ values.

## 1. Introduction

The global warming of the planet and the huge demand for “green” energy with the development of novel materials to be used for energy storage/production applications in a clean, cheap, competitive, safe, and sustainable way is an urgent necessity throughout the world [1]. In this sense, porous silicon oxycarbide-derived carbon (SiOC-DC) materials have shown very promising results to be used as gas storage/capture materials (H_2_ [2,3], CO_2_ [4,5], CH_4_ [2]), electrodes for metal-ion (Li [6], Na [7], etc.) batteries and supercapacitors, etc. [5,8,9,10,11]. Recently, supercapacitors, especially electrical double-layer capacitors (EDLC), based on carbon materials, have received much attention due to their extremely high charge/discharge rates, very long stability (standing up to 1 million cycles during service), low cost, light weight, relatively safety, and high power density (*P_d_*) values [12,13]. However, their principal handicap with respect to ion-Li batteries is their relatively low energy density (*E_d_*) values [11]. Therefore, nowadays, many researchers are trying to increase these values without decreasing both the specific capacitance values (*C_s_*) and long-term stability. The main requirements for the electrode of supercapacitors in order to achieve a great electrochemical response are high electrical conductivity and a selected pore architecture with the presence of micro-, meso-, and macropores [12,13,14]. In addition, the influence of superficial modifications with the incorporation of selected functionalities (N, O, F, etc.) [15,16,17] is also studied. Usually, SiOC materials display a relatively low electrical conductivity, which can be enhanced by increasing both the pyrolysis temperature and the amount of carbon via the addition of vinyl or phenyl silicon precursors [18] and carbon fillers [19,20].

It is important to understand the complex and versatile structure of SiOC materials, which depends on the initial precursors’ compositions, processing conditions, pyrolysis temperature, etc., that allows obtaining a material with a determined composition, structure, and microstructure (i.e., the amount of carbon and porosity) [21]. At low pyrolysis temperatures (≤1200 °C), SiOC materials are composed of a Si-O-C mixed network formed by different units (i.e., SiO_(4−x)_C_x_ where 0 ≤ x ≤ 4) and an embedded homogenously dispersed carbon-free phase (C_free_). At higher temperatures, the SiOC material starts phase separation into SiO_2_ and SiC (both amorphous) and C_free_ (highly disordered). As the temperature increases, the carbon phase undergoes rearrangement into more ordered compounds (i.e., nanocrystalline β-SiC and nano-domains of glassy or turbostratic carbon), but usually SiO_2_ is kept as an amorphous phase [22]. At a temperature ≈ 1400 °C, the material is prone to experiencing carbothermal reduction (1), but its evolution will mainly depend on the composition (amount of carbon), microstructure (porous vs. dense materials), etc. [23,24].

SiO_2_(s) + 3C(s) → SiC(s) + 2CO(g) (1)

There are several methods that can be used to produce highly porous silicon oxycarbide carbon-derived materials (SiOC-DC) with a tuned porosity via the selective removal of SiO_2_/SiC less-conductive phases, which also increases their electrical conductivity [25]. One of them is the halogen (i.e., chlorine) etching route, which has been extensively used with carbide materials giving, as a result, a highly microporous material (0.5–2 nm) due to the metal or semi-metal removal with a very specific surface area (SSA) up to 3300 m^2^ g^−1^ and a high pore volume, which can be adjusted to a sub-Angstrom accuracy via the selection of appropriate carbide materials and etching conditions (i.e., temperature, ratio of gases, etc.) [26,27]. In the case of silicon carbide, this etching can be described by reaction (2). Carbide removal typically maintains the original shape and volume of the initial carbide, and it is known as a conformal transformation process [27]. The removal of SiC generates mainly microporous carbon materials [28].

SiC(s) +2Cl_2_ → SiCl_4_ + C(s) (2)

In the case of SiOC, the presence of different phases within the material (i.e., SiO_(4−x)_C_x_, 0 ≤ x ≤ 4) means that chlorine etching produces hierarchical micro-mesoporous materials. This is due to the existence of the SiC phase, which mainly produces micropores, and the amorphous SiOC phase, which generates a broader pore size distribution of mesopores depending on the SiOC composition (i.e., SiO_(4−x)_C_x_ where 0 ≤ x ≤ 4: SiO_4_, SiO_3_C, SiO_2_C_2_, SiOC_3_, and SiC_4_ units). Initially, Gogotsi et al. [2] assumed that only the mixed bonds of the SiOC matrix could be etched away via chlorine etching according to reaction (3), but later, Tolosa et al. [10] indicated that SiO_2_ bonds can also be extracted via chlorine etching following reaction (4), always with the presence of carbon.

SiO_(4−x)_C_x_ + 2Cl_2_ → (4 − x)/3CO(g) + (4 − x)/3CO_2_(g) + (5x − 8)/3C(s) 0 ≤ x ≤ 4 (3)

SiO_2_(s) + 2C(s) + 2Cl_2_(g) → SiCl_4_(g) + 2CO(g) (4)

As a consequence of the chorine etching of silicon oxycarbide materials, a hierarchical, highly porous carbon microstructure can be obtained where basically micropores come from the SiC phase and broader mesopores (2–10 nm) come from the mixed SiOC network [2].

In this work, we have prepared a C-enriched and highly interconnected SiOC network via pyrolysis of triethoxysilane/dimethyldiphenilsiloxane (TREOS/DMDPS) hybrids easily obtained from sol-gel, which contain both Si-H and Si-Ph bonds [29,30]. After that, SiO_2_/SiC phases have been removed via chlorine etching at a moderate temperature (800 °C) in order to ensure a maximum yield of carbon in the related hierarchical porous SiOC-DC materials [27]. The aim of this work is to elucidate the influence of the pyrolysis temperature (from 1100 to 1400 °C) in the phase separation, the SiOC composition, and the subsequent removal of SiC and SiO(C) phases via chlorine etching, which will render in the formation of a hierarchical porous microstructure comprising micro-, meso-, and macropores. The influence of the pyrolysis temperature of SiOC materials with chlorine etching is a parameter rarely studied in the literature [8,9]. Instead, it employed a selected pyrolysis temperature (i.e., 600 or 1200 °C [4,31,32]), materials with a different composition (i.e., low or high amount of carbon [2]), or different shapes (i.e., fibers [10] or microspheres [11]). For this reason, in this work, we have selected a C-enriched SiOC material pyrolyzed at different temperatures in order to determine the influence of this parameter on the SiOC composition (i.e., SiO_(4−x)_C_x_ where 0 ≤ x ≤ 4: SiO_4_, SiO_3_C, SiO_2_C_2_, SiOC_3_, and SiC_4_ units) and the pore architecture obtained after chlorine etching. In addition, conventional (i.e., lateral size) and non-conventional Raman parameters (i.e., tortuosity, among others) were employed to characterize the C_free_ phase. Finally, the electrochemical performance and cycled stability of the obtained materials were tested in order to evaluate their suitability as electrodes for supercapacitor applications. For the first time, to the best of our knowledge, the microstructure and Raman parameters (conventional and non-conventional) are considered to establish their relationships with the electrochemical response of these SiOC-DC materials.

## 2. Results and Discussion

### 2.1. Characterization of the Hybrid and Tracking of the Pyrolysis Process

**Figure 1:**
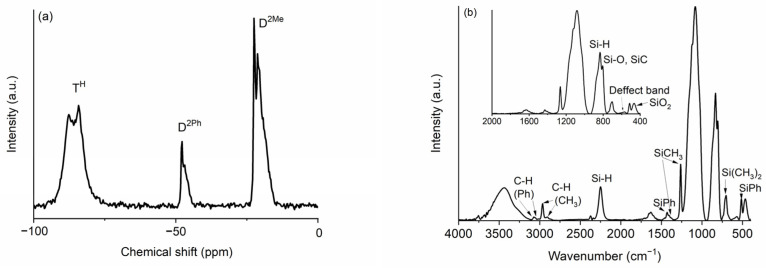
(**a**) ^29^Si MAS NMR and (**b**) FT-IR spectra of the TREOS/DMDPS hybrid.

**Figure 2:**
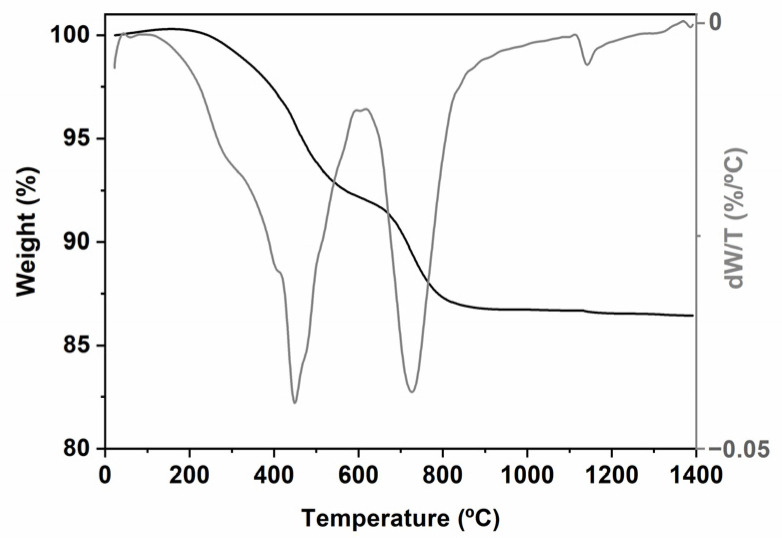
TG/DTG picture of the TREOS/DMDPS hybrid.

The as-prepared TREOS/DMDPS hybrid displays a high amount of carbon (C (%) = 27.3%, Table 1) and a dense microstructure according to N_2_ adsorption/desorption results with both negligible SSA, named *S_BET_*, and porosity (results not shown here). These findings are due to the presence of the high reactivity of TREOS molecules, which produce a highly cross-linked hybrid [30], as well as the low reactivity of DMDPS molecules that enable the incorporation of phenyl groups, increasing the amount of carbon in the dense hybrid [29]. The TREOS/DMDPS hybrid has been characterized by ^29^Si Magic angle spinning nuclear magnetic resonance (^29^Si MAS-NMR) (Figure 1a) and Fourier Transform Infrared (FT-IR) (Figure 1b) spectroscopies and, in addition, its organic to inorganic transformation was followed by thermogravimetric/differential thermogravimetric (TG/DTG) analysis (Figure 2).

The ^29^MAS NMR spectrum of the TREOS/DMDPS hybrid is shown in Figure 1a. There are three groups of signals: T^H^ (≈−90 ppm), D^2Ph^ (≈−50 ppm), and D^2Me^ (≈−20 ppm), which are in accordance with the chemical composition of the precursors; i.e., while TREOS gives T^H^ units, DMDPS gives D^2Ph^ and D^2Me^ ones. The T^H^ units (HSiO_3_) can be tentatively decomposed into T^H^ units copolymerized with the DMDPS polymer (T^H^(D), −88.3 ppm, 17%) and self-condensed (T^H^(T^H^), −84.3 ppm, 32%) [30,33]. This assignation has been made according to the high reactivity of the TREOS molecule and to the low reactivity of the DMDPS polymer, so the self-condensation of TREOS molecules instead of copolymerization with the less reactive DMDPS polymer is favored. The following bands are related to D^2Ph^ units (Ph_2_SiO_2_), with two phenyl side groups of the DMDPS polymer indicating different chemical environments probably ascribed to D^2Ph^ units near D units with and without Ph groups (D^2Ph^-D^2Ph^-D^2Me^ (−47.9 ppm, 2%) and D^2Me^-D^2Ph^-D^2Me^ (−46.8 ppm, 6%)) [34]. The last bands are associated with the D^2Me^ groups (Me_2_SiO_2_) of the DMDPS polymer. A probable assignment in accordance with the percentage calculated is the following: D^2Me^ self-condensed polymer chain units (D^2Me^-D^2Ph^ units (−22.4 ppm, 6%), D^2Me^-D^2Me^ (−21.2 ppm, 14%)) and copolymerized with TREOS, i.e., D^2Me^-T^H^ (−19.3 ppm, 22%). A D^2Ph^/D^2Me^ ratio of 16/84 was found that perfectly agrees with the content of the phenyl groups of the initial composition for the polymer (i.e., 14–18%).

The FT-IR spectrum of the TREOS/DMDPS hybrid (Figure 1b) shows the bands related to the initial precursors, TREOS (i.e., Si-H and Si-O bonds) and DMDPS (i.e., Si-Me, Si-Ph, and Si-O bonds), the copolymerized hybrid, and those related to H_2_O adsorbed during the elaboration of the KBr pellet (3400 and 1640 cm^−1^, stretching and bending of OH, respectively). The bands associated with the Si-H bonds are located at 2250 cm^−1^ (stretching) and 835 cm^−1^ (bending) [33,35]. The bands related to the asymmetric stretching of Si-O bonds related to TREOS and DMDPS and to the copolymerized hybrid appear in the range of 1160–1080 cm^−1^ as a broad band. The symmetric stretching of Si-O bonds appears at 800 cm^−1^ [36]. Another important band is known as the “defect band” (560 cm^−1^), which usually appears in sol-gel organic–inorganic silica-based hybrids due to the steric hindrance of organic groups during the hydrolysis–condensation reactions, giving, as a result, the formation of 4-fold siloxane rings, instead of 6-fold rings during the synthesis of the hybrid, denoting the presence of copolymerized units [36]. Finally, the band located at 460 cm^−1^ is due to the deformation mode of Si-O-Si bonds and indicates a dense and highly cross-linked silica network [37], attributed to the presence of highly reactive Si-H bonds of the TREOS molecule [30]. In the case of methyl groups, the asymmetric and symmetric stretching of C-H bonds appear at 2965 and 2905 cm^−1^, respectively. The asymmetric and symmetric bending of Si-CH_3_ bonds appear at 1400 and 1270 cm^−1^, respectively. A shoulder related to the CH_3_ rocking is observed at 870 cm^−1^, and at 800 cm^−1^, Si-C stretching together with Si-O vibrations is detected. Finally, a peak attributed to O_2_Si(CH_3_)_2_ units is noted at 700 cm^−1^ [38]. In the case of phenyl groups, the asymmetric and symmetric stretching of C-H bonds are located between 3080 and 3050 cm^−1^. The stretching of C=C bonds (1580 cm^−1^) appears to overlap with the bending band of the adsorbed water. The band at 1430 cm^−1^ belongs to the stretching vibration of Si-Ph bonds [38]. At 1120 and 1000 cm^−1^, the bands related to in-plane ring vibrations and bending ring vibration of aromatic rings, respectively, appear [38]. The 510 cm^−1^ peak is associated with Si-C stretching of the Si-Ph bonds [38]. It is important to note that the intensity of phenyl bands related to methyl bands is in accordance with the relative amount of phenyl groups (i.e., 14–18%).

**Figure 3:**
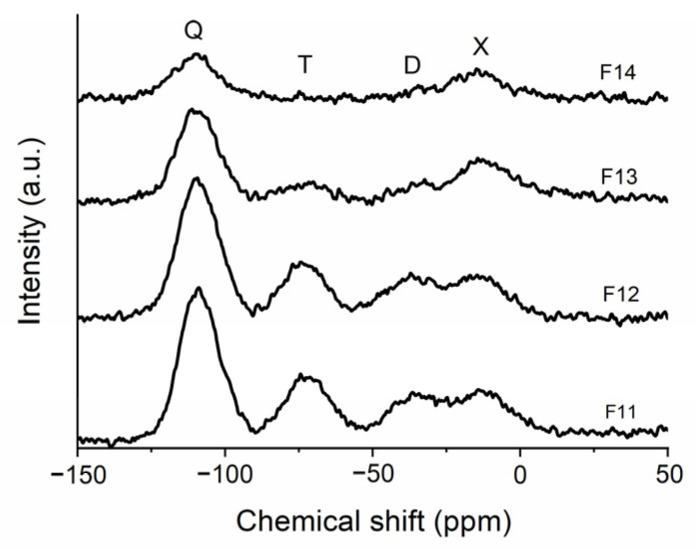
^29^Si MAS NMR spectra of the derived SiOC materials pyrolyzed at different temperatures.

The pyrolysis of the TREOS/DMDPS hybrid was monitored via TG/DTG (Figure 2). The results indicate that there are three regions of weight loss from R.T. up to 350, 600, and 900 °C. From R.T. to 350 °C, a slight weight loss (*WL*) of ≈ 1% is observed. The DTG curve shows a maximum located at 280 °C, related to both the release of solvent-entrapped molecules and hydrolysis/condensation reactions involving residual compounds (i.e., Si-OR/Si-OH groups) [24]. As the pyrolysis temperature increases, several redistribution reactions occur in polysiloxanes between the different groups present (i.e., Si-H, Si-C (i.e., Me and Ph groups), and Si-O) [24,35]. These reactions will occur depending on their reactivity, progressively changing the material, increasing the cross-linking, and, therefore, the thermal resistance of the resulting material. It is important to take into account that the redistribution reactions occur during the thermal process and are responsible for the SiOC composition (i.e., SiO_(4−x)_C_x_ where 0 ≤ x ≤ 4: SiO_4_, SiO_3_C, SiO_2_C_2_, SiOC_3_, and SiC_4_ units). The evolution of these units from the hybrid to the SiOC at different pyrolysis temperatures will be determined via ^29^Si MAS NMR spectroscopy (Figure 3). From 350 to 600 °C, a *WL* of ≈ 7% is observed. According to the high reactivity of the Si-H bonds of the TREOS/DMDPS hybrid, the redistribution reactions associated with Si-H/Si-O bonds mainly occur. These reactions produce the evolution of gaseous species at 445 °C (i.e., mainly SiH_4_) [24,35]. However, in this range of temperatures, the reactions that involve the exchange of Si-C (Me or Ph)/Si-O and Si-O/Si-O bonds can also occur [24]. In the case of linear methyl-siloxane polymers, Si-O/Si-O exchange (i.e., depolymerization reactions) occurs with the formation and evolution of small, cyclic, gaseous oligomers. However, for phenyl-siloxane polymers, the steric hindrance of phenyl groups limits the production of cyclic oligomers and mainly forms benzene via the excision of Si-Ph bonds [39,40]. Finally, from 600 to 900 °C, a *WL* of ≈ 5% centered at 725 °C is detected, mainly ascribed to the organic to inorganic transformation (i.e., mineralization reactions) with the insertion of carbon within the silica network and the formation of the C_free_ phase [24]. The process occurs via a radical mechanism which is promoted by the presence of Si-H bonds [41].

The ceramic yield of the TREOS/DMDPS hybrid is very high (87%), corroborating a highly cross-linked and densified structure of the hybrid network and the moderate evolution of the depolymerization reactions during pyrolysis. A much lower ceramic yield was previously observed in TREOS/polydimethylsiloxane (PDMS) hybrids as a consequence of the great contribution of depolymerization reactions of the linear PDMS polymer during the pyrolysis process [42].

### 2.2. SiOC and SiOC-DC after Chlorine Etching

The evolution of the structure from the hybrid to SiOC was followed by ^29^Si MAS NMR, FT-IR, and X-ray diffraction (XRD). The C_free_ phase was analyzed via Raman spectroscopy, and the microstructure was analyzed via N_2_ adsorption–desorption measurements and field emission scanning electron microcopy (FE-SEM) images. In addition, the transformation on SiOC-CD after halogen etching was followed using the same techniques, excluding ^29^Si MAS NMR due to the low amount of Si sites for the SiOC-CD samples and including high-resolution transmission electron microscopy (HR-TEM) images to complete the microstructural characterization.

The ^29^Si MAS NMR spectra of TREOS/DMDPS-derived SiOC materials pyrolyzed at different temperatures are shown in Figure 3. Each spectrum was deconvoluted, and the chemical shift (ppm) and number of different SiO_x_C_4−x_ sites (%) are collected in Table 2. After pyrolysis, the spectrum radically changes due to the organic to inorganic transformation of the material. The initial environments of the hybrid (T^H^, D^2Ph^, and D^2Me^ (Figure 3)) present in the ^29^Si MAS NMR spectrum disappear, and new ones related to the SiOC network can be observed. The SiOCs pyrolyzed at 1100–1200 °C (i.e., F11 and F12 samples) are composed mainly of Q, T, D, and X units [22] with a fairly similar composition. At these low pyrolysis temperatures, it is important to note the presence of a great amount of X units due to the existence of phenyl groups in the hybrid of origin. At a higher pyrolysis temperature (1300 °C, F13), the reorganization of silicon sites and the phase separation increase the number of both Q and X units, and therefore, T and D units significantly decrease. Finally, only at a pyrolysis temperature of 1400 °C (i.e., F14) only is noticeable the presence of Q and X units (Table 2).

As mentioned above, the evolution of Si sites from the hybrid to the SiOC during the thermal process can be explained by the redistribution reactions (involving Si-H/Si-O and Si-C/Si-O bonds), which continuously change the ^29^Si MAS NMR spectrum. In the earliest stages of the pyrolysis, Si-H/Si-O redistribution reactions mainly occur, which form Q and volatile species (i.e., SiH_4_) and consume the T^H^ units (reaction (5)) [33,35,42]. Later on, different redistribution reactions can form the different Si sites, and their composition basically depends on the initial Si/O ratio (reactions (6)–(8)). In this sense, Q and D units form T units and then M and X units. At higher temperatures, these reactions are no longer at equilibrium and form Q and X units, the most stable Si sites [43].

T^H^ + T^H^ → Q + D^2H^ →→ Q + SiH_4_(g) (5)

Q + D → T(6)

D + T → Q + M(7)

M + T → Q + X(8)

**Figure 4:**
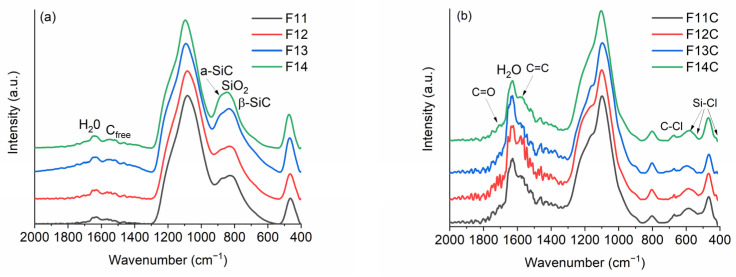
FT-IR of (**a**) SiOC materials pyrolyzed at different temperatures and (**b**) SiOC-DC after Cl_2_ etching at 800 °C.

Due to the organic to inorganic transformation after pyrolysis, the FT-IR spectra (Figure 4a) radically change. The bands related to Si-H, Si-CH_3_, and Si-Ph totally disappear, and only the bands related to the SiOC mixed network and C_free_ appear [44]. The SiOC displays the bands associated with silica-derived materials located at 1082 and 461 cm^−1^ (asymmetric stretching of Si-O and bending mode of Si-O-Si, respectively), which shift to higher wavenumbers (1095 and 471 cm^−1^, respectively), as the pyrolysis temperature increases due to the strengthening and densification of the SiOC network [37]. The stretching of the C=C bonds related to the C_free_ phase appears as a broad band around 1540 cm^−1^ besides the one related to H_2_O. In the 900–700 cm^−1^ spectral range, the stretching bands of Si-O and Si-C bonds, which are steadily modified as the pyrolysis temperature increases, appear overlapped. The samples pyrolyzed at 1100 and 1200 °C display a band related to the mixed SiOC network at 826 cm^−1^ (SiX_4_ X = O, C) [44] and a shoulder related to amorphous SiC at 872 cm^−1^ (a-SiC). At higher temperatures, the phase separation of SiOC occurs, producing the formation of SiO_2_ and SiC and a rearrangement that partially transforms the a-SiC into nanocrystalline β-SiC (780 cm^−1^) [45,46]. As a result, in this range of temperatures, this band experiences a broadening and a shifting to lower wavenumbers. A similar behavior has been previously observed in related materials [29,42].

As expected, after Cl_2_ etching, the bands related to the SiOC network (both SiO and SiC) are drastically reduced as a consequence of the removal of SiC and SiO bonds (Figure 4b). However, in all cases and independently of the pyrolysis temperature, the bands related to silica (1097, 803, and 467 cm^−1^) can be observed, indicating that it is not totally removed and, of course, the band related to C=C bonds (1570 cm^−1^) of the C_free_ phase can now be clearly observed. In addition, some new bands appear as shoulders located at 1210–1230 and 1710–1750 cm^−1^, probably related to the formation of C=O functionalities (C=O and C-O stretching, respectively). Some authors assigned the C=O functionalities to lactone, anhydride, and carboxylic groups [4,31,32]. At 671, 590, 524, and 425 cm^−1^, some small bands related to C-Cl (800–600 cm^−1^) [31] and Si-Cl (625–425 cm^−1^) [47] bonds appear with different amounts of chlorine related to some residual moistures after etching and post-treatment in H_2_. It is important to note that these bands display small intensities, indicating the low residual amount of chlorine in the prepared SiOC-DC materials. The presence of C=O and C-Cl functionalities have been previously observed in SiOC-DC materials etched with Cl_2_ [4,31,32].

**Figure 5:**
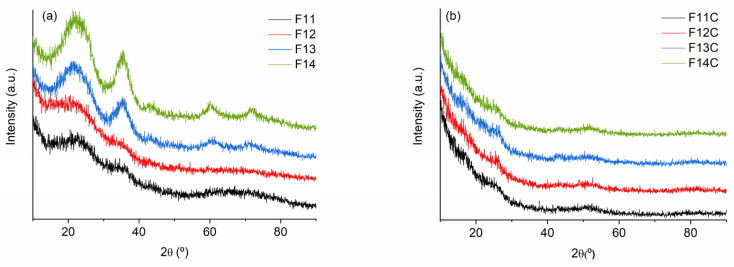
XRD patterns of (**a**) SiOC materials pyrolyzed at different temperatures and (**b**) SiOC-DC materials after Cl_2_ etching at 800 °C.

The XRD patterns of SiOC samples pyrolyzed at 1100–1200 °C are almost featureless, showing the bands related to amorphous silica-derived materials (broad halo 2θ ≈ 22°) and β-SiC (2θ ≈ 35, 60 and 75° related to (111), (220) and (311) lattice planes, respectively; JCP: 00-029-1129). The presence of carbon of the C_free_ phase can also be observed as a broad halo 2θ ≈ 26°, related to ordered graphite ((002) JCP: 00-041-1487)), and another one at 2θ ≈ 43–44° (10), associated with disordered carbon or graphene-like carbon materials without stacking order [48,49]. The intensity of these bands is enhanced with the pyrolysis temperature (1300–1400 °C) as a consequence of the phase separation, the subsequent rearrangement and an incipient crystallization of the amorphous carbon phases (i.e., SiC and C_free_) [22] is observed (Figure 5a)_._ A similar response has been previously observed in related SiOC materials [29,42].

After the chlorine etching, independently of the pyrolysis temperature, the bands associated with SiO_2_ and SiC have almost disappeared, and only the bands related to carbon can be slightly observed (Figure 5b). The absence of a sharp peak located at 2θ ≈ 26° (002) and the presence of a broad band at 2θ ≈ 43–44° (10) could indicate the amorphous/disordered structure of SiOC-DC materials with the lack of AB stacking order between the graphene layers [48].

**Figure 6:**
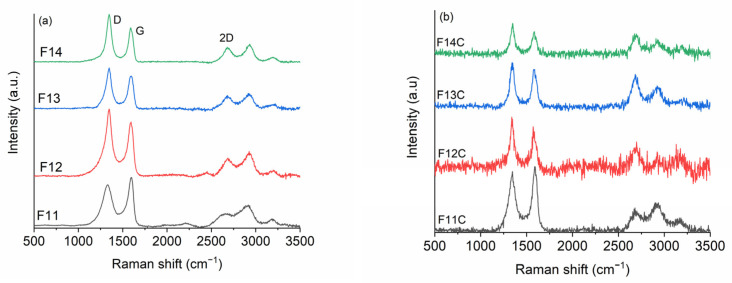
Raman spectra of (**a**) SiOC and (**b**) SiOC-DC materials, respectively, pyrolyzed at different temperatures.

Based on FT-IR and XRD results, the prepared SiOC-DC materials are basically composed of carbon, and therefore, we have also employed Raman spectroscopy to fully investigate the C_free_ phase evolution during pyrolysis and the evolution of SiOC to SiOC-CD materials after Cl_2_ etching. It is well known that Raman spectroscopy is a very powerful technique that can be used to determine the most important characteristics of the C_free_ phase of SiOC materials. The main bands are D (1360 cm^−1^) [50] and G (1580 cm^−1^) [51], mainly assigned to disorder and order in carbon materials, respectively. The second-order bands, 2D (2700 cm^−1^), D + G (2950 cm^−1^), and 2D’ (3250 cm^−1^ [52], sometimes play an important role. In this sense, the intensity ratio between *I_D_/I_G_* and *I*_2*D*_/*I_G_*, the appearance of some bands, and, of course, their position, width, and intensity of some bands fully describe the characteristics of carbon materials [51,53,54,55,56]. The in-plane length of carbon crystallites of nanoclusters (*L_a_*) [51,56] is inversely proportional to the ratio of D and G bands, and largely, it is the most common parameter. Continuous graphene length, including tortuosity (*L_eq_*) [57], the average distance between defects (*L_D_*) [55], and 3D-ordering of the graphene layers (Δ*W*_2*D*_^−1^) [53] are other less common parameters. These Raman parameters have started appearing in recent literature for SiOC-derived materials [20,58,59,60,61]. However, to the best of our knowledge, it is the first time that these parameters have been employed in SiOC-CD materials. Figure 6 indicates the Raman spectra of (a) SiOC and (b) SiOC-DC materials, respectively, pyrolyzed at different temperatures. Following the same criteria employed in previous work [58], a single Lorentz fitting was performed over the Raman spectra, and the calculated derived parameters are shown in Table 3. The values included in Table 3 are the position (cm^−1^), full width at half intensity (*W*) (cm^−1^) of the D, G, and 2D bands; *L_a_* (nm); *L_eq_* (nm); *L_D_* (nm); ∆*W*_2*D*_^−1^ (cm); and the *I*_2*D*_/*I_G_* ratio.

In the case of SiOC materials, the SiOC sample pyrolyzed at 1100 °C displays a Raman spectrum characteristic of disordered carbons with broad and overlapped D and G bands but with the presence of second-order bands (i.e., 2D, D + G, and 2D’). As the pyrolysis temperature increases, all bands, especially D and 2D, become narrower and upshifted (Table 3), indicating an ordering rearrangement from disordered to glassy carbon [54]. The *I_D_/I_G_* ratio increases with the pyrolysis temperature, and oppositely, *L_a_* decreases from 5.1 to 3.3 nm (Table 3). This behavior must be mainly due to the ordering degree experienced with a small contribution of the carbothermal reduction of silica, considering the rather low variation in the carbon content from 1100 to 1400 °C (Table 4). It is also observed that both ∆*W*_2*D*_^−1^ and *I*_2*D*_/*I_G_* values follow the same trend, which corroborates this assumption. In addition, *L_D_* decreases from 12.2 to 9.7 nm, indicating a more defective C_free_ phase as the pyrolysis temperature increases. This fact is in consonance with a previous work [62] that indicated that the C_free_ phase formed during the pyrolysis (i.e., of a preceramic material) is included within the Si-O-C network. As a result, high local strains are generated over the graphene layers, increasing the disorder as the pyrolysis temperature increases. Finally, the values of *L_a_* and *L_eq_* are similar (*L_a_* ≈ *L_eq_*) independently of the pyrolysis temperature (Table 3), indicating that the graphene layers are planar with a low quality of interconnection [55].

The Raman spectra of the samples after the chlorine etching suffer deep changes. The luminescence background is enhanced, and the resulting spectra are also much noisier. This fact can be somehow related to the presence of dangling and broken bonds formed during the removal of the Si-related nano-domains after chlorine etching. In all cases, the amount of carbon radically increases and is rather similar, independently of the initial pyrolysis temperature, as can be seen in Table 3.

The chlorine etching radically influences the C_free_ phase, *W_D_* and *W*_2*D*_ are drastically reduced, *L_a_* and *L_D_* remain practically similar with respect to the related SiOC, but the *L_eq_*, ∆*W*_2*D*_^−1^, and *I*_2*D*_/*I_G_* parameters are deeply enhanced for the samples pyrolyzed at temperatures ≥ 1200 °C (Table 3). *L_eq_* values are approximately three times higher than *L_a_* (*L_eq_* > > > *L_a_*) for the samples pyrolyzed at temperatures > 1200 °C, indicating the presence of well-interconnected tortuous graphene layers. In addition, the great enhancement experienced by ∆*W*_2*D*_^−1^ and *I*_2*D*_/*I_G_* parameters indicates a high quality of crystallinity in the graphene layer at curvature. In agreement with Larouche et al. [57], the greater ∆*W*_2*D*_^−1^, the greater the quality of the crystallinity, so higher values of both *L_eq_* and ∆*W*_2*D*_^−1^ indicate a higher quality of crystallinity of the tortuous graphene layers.

In summary, F11C displays the biggest and least defective C_free_ domains (biggest *L_a_* and *L_D_*, Table 3). These carbon domains are planar with a low degree of interconnection and crystallinity. At higher temperatures, especially noticeable for F13C and F14C, the C_free_ domains are smaller and more defective (lower *L_a_* and *L_D_,* Table 3), but they show the highest tortuous domains with both the highest degree of interconnection and crystallinity.

The evolution of the microstructure from SiOC to SiOC-CD materials has been followed by means of N_2_ adsorption–desorption measurements and FE-SEM images. SiOC materials pyrolyzed at different temperatures display a dense microstructure showing type II isotherms, very low *S_BET_* values, and almost negligible porosity in accordance with their pore size distribution (PSDs). It is noticeable that the F12 sample displays a type IVa isotherm with a PSD that indicates a small number of mesopores and a slightly higher *S_BET_* value (Figure S1a,b and Table 4). The F12 isotherm has a type H3 hysteresis loop, which could be related to the presence of some macropores [63]. The FE-SEM micrographs are in perfect agreement with these results. F11 (Figure S2a) is a dense material but shows an inhomogeneous surface, F12 (Figure S2b) displays a slightly porous surface, and the surface of F13 and F14 (Figure S2c,d) looks very different, probably ascribed to the phase separation of SiOC and the ulterior crystallization of β-SiC, which is enhanced with the temperature in accordance with both FT-IR (Figure 4) and XRD (Figure 5) results.

**Figure 7:**
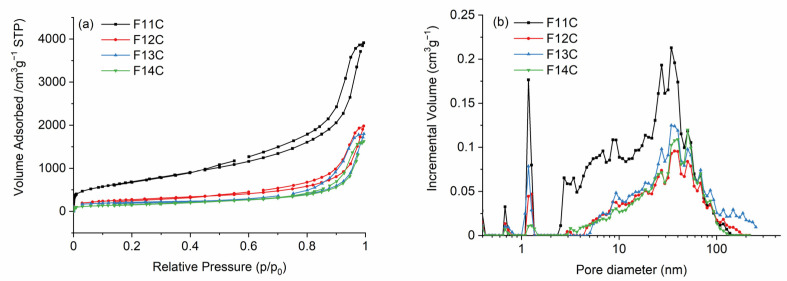
(**a**) N_2_ adsorption–desorption isotherms and (**b**) pore size distribution of SiOC-DC materials pyrolyzed at different temperatures after Cl_2_ etching.

After Cl_2_ etching, the texture radically changes, and the removal of the SiOC mixed network and/or SiO_2_ and SiC species after phase separation generates a hierarchical SiOC-DC material composed of micro-, meso-, and macropores. In all cases, the isotherms are type IVa (Figure 7a) and related to the presence of both micro and mesopores. The steep uptake at very low p/p_0_ values resembles type Ib isotherms characteristic of materials containing wide micropores and small mesopores of around <2.5 nm [63]. In addition, they have type 3–4 hysteresis loops, indicating the contribution of macropores and characteristic of micro-mesoporous carbon materials, which can have slit-like shape pores. All of these features indicate a complex and broad PSD, which perfectly agrees, with the PSD observed in Figure 7b. *V_SP_* values, determined by the DFT model, are somewhat higher than *V_tot_* values since the PSD curve obtained by DFT is calculated taking into account the variation of the surface tension of adsorbed N_2_ molecules as a function of the pore size, while *V_SP_* is directly calculated at p/p_0_ = 0.99 from the adsorbed volume on the measured N_2_ isotherm.

SiOC pyrolyzed at the lowest temperature is composed of an interconnected and dense SiOC network (Table 2), which initially slows down the Cl_2_ etching of the SiOC-SiO_2_ enriched units (Q, T, and D units, Table 2), and instead, due to the very aggressive etching conditions, the SiOC-SiC enriched units (particularly X units, Table 2) are preferentially removed (2), generating a great amount of micropores. Later on, the etching continues, generating larger meso- and macropores due to the removal of bulkier SiOC species (Q, T, and D, Table 2) in accordance with (3) and (4). The latter equation is promoted by the high amount of carbon in the initial SiOC samples (Table 4). The obtained SiOC-DC materials display the highest *S_BET_* value due to the presence of the greatest amount of micropores but also contain the largest amount of both meso- and macropores. It is important to notice that due to the very strong Cl_2_ etching conditions, the amount of porosity generated is much higher than previously reported when HF etching was employed in related SiOC materials [16,29] and is comparable to SiOC-DC materials obtained via Cl_2_ etching (with *S_BET_* of 3089–2227 m^2^ g^−1^ [10]). In the case of HF etching, the nucleophilic attack is performed only over polarized Si-O bonds, but in the case of Cl_2_ etching, the attack is initially experienced by the Si-C bonds and then over SiO(C) bonds. As a result, a more porous microstructure is obtained with Cl_2_ etching. As the pyrolysis temperature increases, the SiOC starts decomposing into SiO_2_ and SiC, and the etching has no restrictions; the average mesopore size is significantly enlarged from 10 to 17 nm (Table 4). The SiOC suffers an unselective removal of the mixed SiOC network, but also of SiO_2_ and SiC bonds, preferentially formed during the phase separation (Figure S2c,d). The F12 sample is still composed of a SiOC mixed network but has an initial slightly porous pristine microstructure that facilitates Cl_2_ etching, generating a great number of macropores and fewer micro and mesopores compared with the F11 sample (Table 4). F13 and F14 samples, which are totally phase-separated, experience a massive removal of both SiC and SiO_2_ units, generating a hierarchical porous microstucture of small (mainly micro and meso) pores which are progressively enlarged as the phase separation progresses, directly related to the size of the SiC domains. All of these facts give as a result a hierarchical PSD composed of wider micro-, meso-, and macropores, which produces a decrease in the volume of all type of pores (Table 4). The decrease in the size of *L_a_* observed in the C_free_ phase of these samples (Table 3) could facilitate etching with Cl_2_ (4) by increasing the active sites susceptible to the Cl_2_ attack.

**Figure 8:**
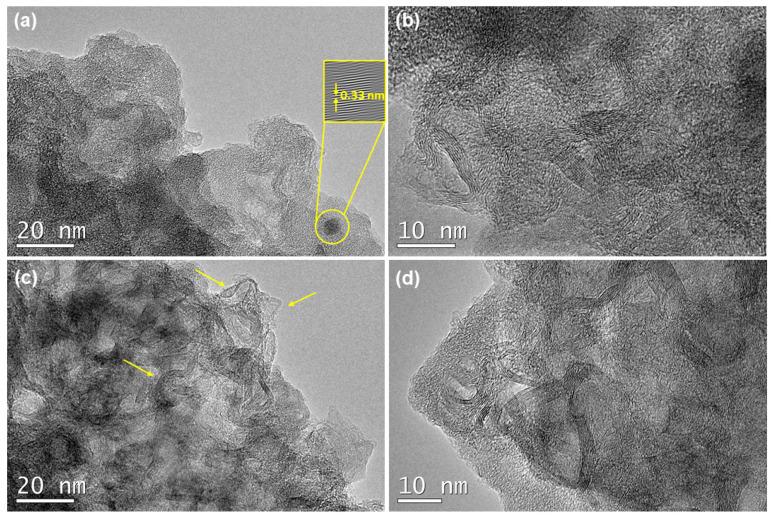
HR-TEM images of SiOC-DC materials pyrolyzed at different temperatures. (**a**,**b**) F11C and (**c**,**d**) F14C. (**a**) Feature of an isolated carbon domain of F11C sample. The top right inset corresponds to Inverse Fast Fourier Transform (IFFT) image used to measure the d-spacing (d = 0.33 nm). (**b**) Superimposed homogeneous carbon domains. The C_free_ phase of F14C sample is composed of (**c**) highly curved inhomogeneous carbon domains (pointed by arrows) and (**d**) highly interconnected carbon domains.

Finally, the HR-TEM images were employed to complete the microstructural characterization of the SiOC-DC materials, which show very different features depending on the initial pyrolysis temperature. The F11C sample displays disordered carbon ribbons (i.e., C_free_ phase) surrounded by the remaining SiOC matrix, according to FT-IR results (Figure 4). In addition, an isolated planar carbon domain can be observed (Figure 8b, d-spacing = 0.33 nm related to (002) lattice plane (JPC: 01-075-1621)). A higher magnification image (Figure 8b) mainly shows straight carbon ribbons with fairly similar and homogeneous carbon domains (around eight graphene layers) superimposed but poorly connected. However, the F14C sample shows a microstructure totally different. The lower magnification image displays inhomogeneous but more highly ordered tortuous carbon domains embedded in the remaining SiOC matrix that show a very different amount of graphene layers varying from a couple to 6–8 layers (Figure 8c, indicated by arrows). This result can be due to the presence of defects originating from a higher pyrolysis temperature and the subsequent Cl_2_ etching. These features can be more clearly observed in a higher-magnification image (Figure 8d), where a high interconnection can be appreciated through curvature and in-plane defects such as vacancies. The presence of pores can also be noticed in both samples. Previous studies indicated the presence of curved carbon (both single graphene sheets and graphite) in SiOC-DC obtained at the pyrolysis temperature of 1200 °C after chlorine etching [2]. It is important to note that these results are not only in agreement with Raman findings but also corroborate them. The C_free_ of the F11C sample is planar (*L_a_* = *L_eq_*) with a low degree of interconnection and the highest and least defective one (the highest *L_a_* and *L_D_* values). In addition, the C_free_ related to the F14C sample presents highly tortuous carbon domains with both a high degree of interconnection (*L_a_* < < < *L_eq_*) and crystallinity (the highest Δ*W2D*^−1^ and *I*_2*D*_/*I_G_* values).

### 2.3. Electrochemical Characterization

Several studies of carbon-derived materials obtained via chlorine etching have indicated the suitability of this type of material for electrochemical applications, especially in the field of electrodes for supercapacitors [8,9,10]. The main requirements are enough electrical conductivity; the presence of functionalities (O, N, etc.) that increase the wettability of the electrolyte, leading to an effective mass transfer; and a tuned microstructure, which comprises the presence of micro-, meso-, and macropores [13]. It was first supposed that only micropores are necessary to achieve high *C_s_* values [9]. However, recent studies indicated that the presence of large and narrow micropore domains can reduce the *C_s_* values, especially at high current values, because they cannot produce contact between the electrolyte and the surface of the electrode [15]. The recent trend includes the design of hierarchical porous carbon materials with the presence of bigger meso- and macropores, which act as pathways, improving the ion transportation and enhancing the availability of micropores via the electrolyte, increasing their electrochemical response (*C_s_*, *P_d_*, and *E_d_*) [12,14,15].

In this work, the SiOC-DC materials obtained via chlorine etching display different features depending on the initial pyrolysis temperature, which are especially important in the case of the microstructure (pore size and amount of pores, Table 4) and C_free_ phase (Table 3), which will for sure influence their electrochemical response.

**Figure 9:**
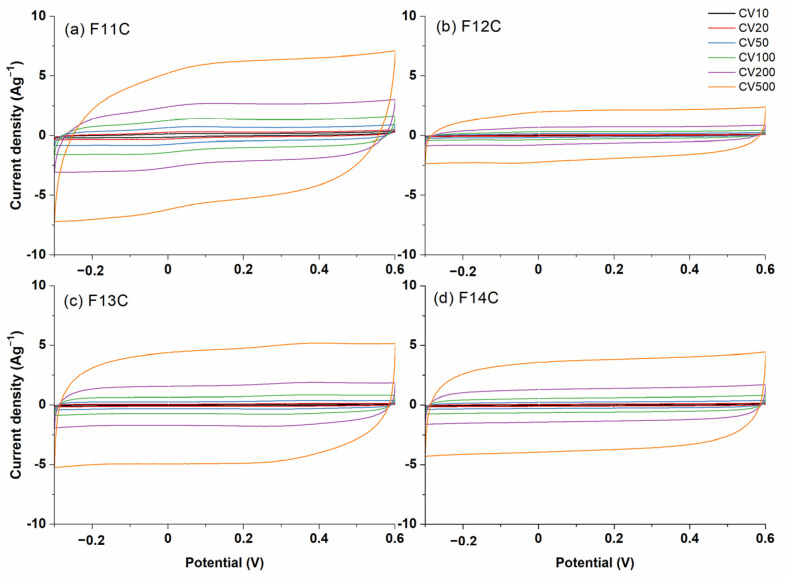
CV curves at scan rates ranging from 10 to 500 mVs^−1^ of SiOC-DC materials after Cl_2_ etching pyrolyzed at different temperatures (**a**) 1100 °C, (**b**) 1200 °C, (**c**) 1300 °C, and (**d**) 1400 °C, respectively.

The CV curves (from 10 to 500 mVs^−1^) for the SiOC-DC materials pyrolyzed at different temperatures after Cl_2_ etching are represented in Figure 9. As can be clearly seen, in all cases, the SiOC-DC materials display CV curves with an almost rectangular shape, indicating an electrochemical double-layer capacitance behavior up to 1000 mVs^−1^ (Figure S3a) resembling the quick formation of a double layer, even at high rates with pseudo-capacitance contribution. These features corroborate the low amount of C=O and C-Cl functionalities found by FT-IR in their spectra (Figure 4). The area of the CV curves directly related to the *C_s_* values increases with the scan rate (Figure 9) but not with the pyrolysis temperature, as it was previously observed in HF-etched SiOC-DC materials [16,29]. The maximum values of *C_s_* are obtained for the F11C sample (i.e., SiOC-DC material pyrolyzed at 1100 °C after Cl_2_ etching), which displays a hierarchical microstructure with the highest values of *S_BET_* and the largest amount of micro-, meso-, and macropore volumes (Table 4). In addition, this sample shows the largest and least defective carbon planar domains without the presence of tortuosity (Table 3), as it has been shown in HR-TEM (Figure 8a,b).

**Figure 10:**
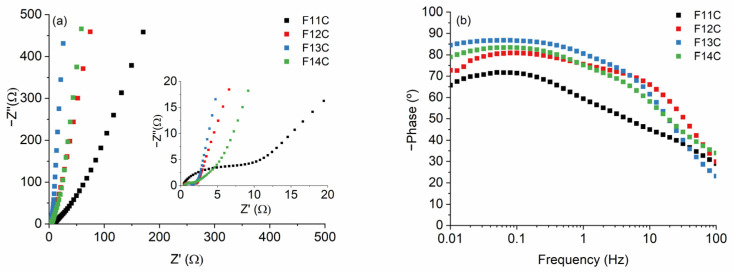
(**a**) Nyquist plots (the inset on the right shows an enlargement of the figure) and (**b**) Bode plots for the SiOC-DC materials pyrolyzed at different temperatures after Cl_2_ etching.

The typical Nyquist plots composed of a semicircle and a vertical line at high and low frequencies, respectively, are collected in Figure 10. The equivalent series resistance (*R_ES_*), due to the internal resistance of the electrode and the electrolyte, are low and quite similar in all cases (Table 5). This indicates a correct two-electrode system setup configuration. The charge transfer resistance at the electrode/electrolyte interface (*R_CT_*) quickly decreases from F11C to the other samples (Table 5). As a first approximation, we only considered that this fact is mainly due to the degree of order determined by the *I*_2*D*_/*I_G_* Raman parameter, directly related to the electrical conductivity of the C_free_ phase. The *I*_2*D*_/*I_G_* values increase with the pyrolysis temperature from 0.27, 0.57, 0.78, and 0.86 for F11C, F12C, F13C, and F14C, respectively (Table 3). The quasi-vertical line at low frequencies also indicates the ideal capacitive behavior of these samples. The lines are steeper as the pyrolysis temperature increases, which again can be associated with the increase in the electrical conductivity of the samples (i.e., C_free_ phase) and, therefore, a faster kinetic reaction. The high values of the *Bode* plots (Figure 10b) indicated the ideal capacitive behavior of the samples, which is in accordance with the shape of CV curves (Figure 9). The time constant (*τ*_0_ calculated at a frequency of ≈ 45 °, where *τ*_0_ *= 1/f*_0_) radically decreases from the F11C to the rest of the samples, again probably ascribed to the higher electrical conductivity of the samples pyrolyzed at higher temperatures. It is important to point out that the value obtained for the F11C sample is slightly lower than those obtained by SiOC-DC after HF etching [16] but also similar to the SiOC-DC materials obtained via Cl_2_ etching [8,9,10].

**Figure 11:**
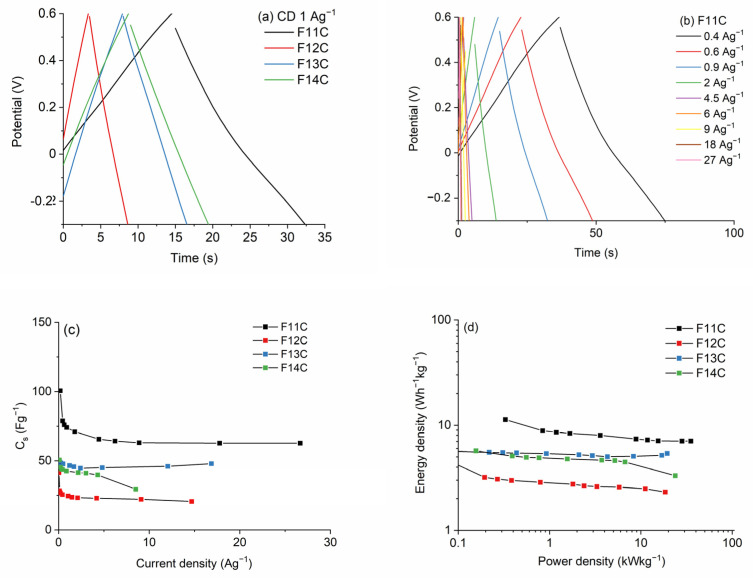
(**a**) GCD curves of SiOC-DC materials pyrolyzed at different temperatures after Cl_2_ etching at a current density of 1 Ag^−1^, (**b**) GCD curves for the F11C sample, (**c**) C_s_ values, and (**d**) Ragone plots, respectively.

The GCD curves at 1 Ag^−1^ of the SiOC-DC materials pyrolyzed at different temperatures after chlorine etching are collected in Figure 11a. The quasi-symmetrical triangular shape indicates the typical double-layer capacitive behavior, in agreement with CV and EIS curves. In the case of the F11C (Figure 11b) material, the high retention ratio as the current density increases up to 27 Ag−^1^ indicates a high rate capacity of this sample. These values are sustainably reduced in the SiOC-DC materials pyrolyzed at higher temperatures (Figure S3b–d). The *C_s_* values calculated by the galvanostatic discharge values for all SiOC-DC materials are depicted in Figure 11c. In accordance with CV curves, the highest values are obtained for the sample F11C, which, in terms of microstructure, is the sample with the largest values of *S_BET_* and the largest amount of micro-, meso-, and macropore volumes (Table 4). With respect to the C_free_ phase, this sample displays the largest and least defective carbon planar domains (Table 3). At a low current density of 0.2 Ag^−1^, F11C displays a *C_s_* value of 101 Fg^−1^. This value decreases up to 63 Fg^−1^ as the current density increases up to 10 Ag^−1^ and remains constant at the very high value of 27 Ag^−1^. These values are similar to or higher than those previously reported for other different SiOC-DC materials also obtained via Cl_2_ etching [8,9,10,64] but employing different experimental and electrochemical measurement conditions. Previously prepared materials show comparable or similar *S_BET_* values but without the presence of pores bigger than 15–20 nm (i.e., meso/macropores). The presence of a great volume of these bigger pores (i.e., meso/macropores > 15–20 nm, Table 4) could explain the good electrochemical response of the F11C sample. Obviously, this sample shows the highest volume of all types of pores, so this is in accordance with the highest *C_s_* values observed. As Sevilla et al. [65] indicated, the presence of bigger pores (i.e., meso/macropores) can produce the formation of channels that promote ion transportation to the lower-size pores (i.e., micropores), which are less accessible, enhancing *C_s_* values. In addition, the C_free_ phase cannot be ruled out, and, in this sense, F11C is composed of the largest and least defective carbon domains, which also play a positive role in the *C_s_* values. On the contrary, better kinetic behavior is observed for the other samples pyrolyzed at higher temperatures (i.e., F12C, F13C, and F14C) that display a high degree of order and, therefore, a higher electrical conductivity (i.e., lower impedance values, Figure 10a). However, this effect is attenuated by the huge decrease in the *S_BET_* and by the micro-, meso-, and macropore volumes (Table 4).

**Figure 12:**
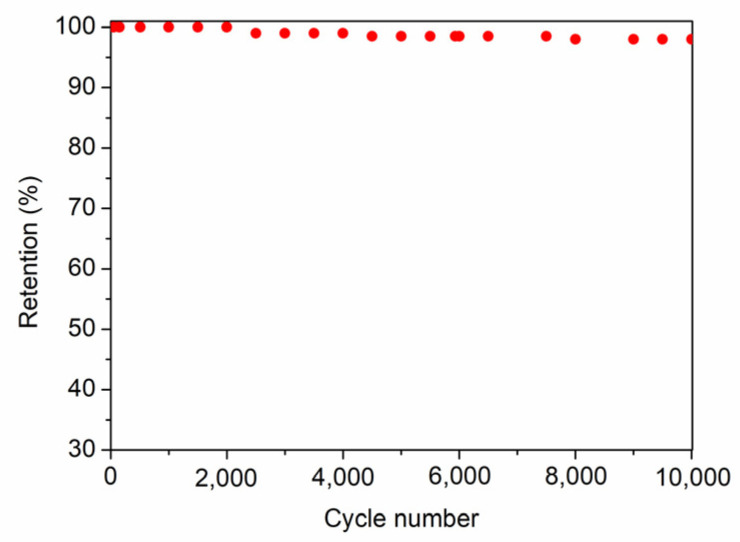
Cycling performance at 0.5 Ag^−1^ for the cell built with F11C.

As can be clearly seen in Ragone plots and in accordance with all data exposed above, the F11C sample shows the highest values of E_d_ (12 and 7 Wh^−1^ kg^−1^) at both low and high P_d_ values (0.32 and 35 kW kg^−1^). These values are slightly lower than those obtained for SiOC-DC materials employing ionic liquids as electrolytes, with a much wider operational window (≥2 V) [8,11,14] than that of the aqueous electrolyte employed in this work (H_2_SO_4_ 1M, 0.9 V). This highlights the value of the results obtained in this study. The cycling performance, which was evaluated for the cell constructed with F11C over 10.000 cycles, shows 98% retention of its initial capacitance (Figure 12).

## 3. Materials and Methods

### 3.1. Experimental Procedure

The TREOS/DMDPS hybrid was synthesized via the sol-gel method using TREOS ((SiH(OCH_2_CH_3_)_3_; 97% (ABCR, Karlsruhe, Germany)) and silanol-terminated DMDPS (OH–Si(CH_3_)_2_–O-[Si(CH_3_)_2_–O]_n_[Si(C_6_H_5_)_2_–O]_m_-Si(CH_3_)_2_OH (ABCR, Karlsruhe, Germany)). The TREOS/DMDPS weight ratio was 60/40, and the molar ratio of isopropanol (i-PrOH, for analysis (Merck, Darmstadt, Germany)), water and hydrochloric acid (HCl, 37% (Merck, Darmstadt, Germany)) were TREOS/i-PrOH/H_2_O/HCl = 1/6/3/0.03. The SiOC materials were obtained via pyrolysis under N_2_ (150 cm^3^/min) in the temperature range from 1100 to 1400 °C, with a heating/cooling rate of 5 °C/min and a dwelling time of 1 h. The SiOCs were grounded employing an agate mortar, and then the powders were sieved below 100 μm. Subsequently, chlorine etching was performed with SiOC powders (1 g). The sample was heated until 800 °C under N_2_ (50 cm^3^/min) at a heating rate of 5 °C/min, and when this temperature was reached, chlorine started to pass through (20 cm^3^/min) for 3 h. After that, the flow of chlorine was stopped, and the sample was cooled only under N_2_ atmosphere (50 cm^3^/min) at 20 °C/min. A final treatment under H_2_ at 800 °C for 3 h was performed to remove the remaining chlorine in the SiOC-DC materials. The samples were denoted as F (for the hybrid material), Fxx (for SiOC materials), or FxxC (for SiOC-DC materials), where F corresponds to TREOS/DPDMS, xx indicates the pyrolysis temperature (11 = 1100 °C, 12 = 1200 °C, 13 = 1300 °C and 14 = 1400 °C), and C indicates the chlorine etching.

### 3.2. Material Characterization

The chemical composition of the as-prepared hybrid and SiOC materials was estimated by both the C (%) and O (%) contents employing the elemental analyzers CS-200 and TC-500 (Leco Corp., Benton Harbor, MI, USA). The Si (%) was calculated by difference from 100%. In the case of SiOC-DC materials, the C (%) was estimated by the weight loss of the TG analysis under flowing air at 10 °C/min until 1200 °C.

The structural evolution from hybrid to SiOC and SiOC-DC was followed using several techniques. ^29^Si MAS-NMR spectra were collected using a 4 mm triple channel probe with a Bruker AV-400-WB spectrometer operating at Larmor frequency of 79.46 MHz. Powdered samples (i.e., hybrid and SiOC) were packed in ZrO_2_ rotors with a Kel-F cap at R.T and spinning rate of 10 kHz. ^29^Si MAS NMR spectra were collected employing a single π/3 pulse at 60 KHz with a recycle delay of 60 s over a spectral width of 40 kHz. Approximately 900 s free induction decays were collected and averaged to obtain each spectrum. Kaolin signal (91.2 ppm) was used as secondary reference relative to tetramethylsilane (TMS) employed as primary reference. The conventional notation of the chemical environments of the Si site is employed here, where Q = [SiO_4_], T = [CSiO_3_], D = [C_2_SiO_2_], M= [C_3_SiO], X = [C_4_Si]. The subscript indicates the number of bridging O atoms. The superscript H corresponds to the number of H atoms replacing C atoms, the superscript Ph or Me indicates the number of these groups present in Si units, and, in the case of copolymerized units, it is indicated by brackets. FT-IR was used for hybrid, SiOC, and SiOC-DC materials employing a Spectrum BX apparatus (PerkinElmer Corp., Waltham, MA, USA) with a resolution of 4 cm^−1^ in the transmission mode using the KBr pellet method. XRD analysis was conducted over SiOC and SiOC-DC materials using a D8 Advance (Bruker, Billerica, MA, USA) apparatus using a Cu k_α_ radiation (λ = 0.154178 nm) in the range of 10 ≤ 2θ ≤ 90^◦^ by steps of 0.05^◦^ and an acquisition time of 1.5 s per step. The pyrolysis process (i.e., from R.T. to 1400 °C) from hybrid to SiOC was followed employing TG and the related DTG curves under flowing N_2_ at 10 °C/min with an SDT Q600 apparatus (TA Instruments, New Castle, DE, USA). Raman spectra of the *C*_free_ phase present in the SiOC and SiOC-DC materials were obtained with an InVia Raman spectrometer (Renishaw plc., New Mills Wotton-under-Edge Gloucestershire, UK) equipped with a 514 nm Ar^+^ laser and calibrated with the intense silicon peak located at 520 cm^−1^. N_2_ adsorption–desorption experiments were carried out at −192 °C using a Tristar 3000 in the case of hybrid and SiOC materials and a more accurate ASAP 2020 apparatus (both of Micromeritics Corp., USA) in the case of SiOC-DC materials. The samples were previously degassed at 120–180 °C during 24 h. *S_BET_* was evaluated using Brunauer–Emmet–Teller equation (BET) [66], employing the adsorption data in the partial pressure (p/p_0)_ range from 0.05 to 0.20. BET plots are included in Figure S4a–d with their corresponding correlation coefficients to show that the p/p_0_ applied range is correct. In the case of SiOC samples, the pore size distribution (PSD) in the mesoporous range (2–50 nm) and average mesopore diameter (*D*_meso_) were obtained from the adsorption branch of the isotherm using the Barrett–Joyner–Halenda equation (BJH) [67]. Finally, the density functional theory (DFT) [68] was used for determining the volume of micropores *V_micro_* (<2 nm), mesopores *V_meso_* (2–50 nm), macropores *V_macro_* (>50 nm), and total volume of pores (*V_tot_)*. The volume of pores determined at p/p_0_ = 0.99 (i.e., single point = *V_SP_*) was also used. FE-SEM S-4700 (Hitachi, Ltd., Tokyo, Japan) operating at 20 keV was used to complete the microstructural characterization of fresh fracture of the SiOC materials and, finally, HR-TEM operating at 200 kV JEM 2100 (JEOL, Ltd., Tokyo, Japan) was employed for studying the microstructure of the C_free_ phase of a selection of SiOC-DC materials. The interplanar distances of selected crystallized zones were determined using Fast Fourier Transform (FFT) and IFFT employing a Gatan software DigitalMicrograph and elucidated in accordance with the d-spacing of the XRD JPD patterns.

### 3.3. Electrochemical Measurement

The electrochemical characterization of the SiOC-DC materials was completed using a symmetrical two-electrode home-made Swagelok^®^-type cell using stainless steel current collectors and employing 1 M H_2_SO_4_ solutions as electrolyte. For the working electrode, approximately 5 mg of active material (SiOC-DC) was mixed in an agate mortar with carbon black as a conductive agent (CB, EnsacoTM E250G, Timcal, Imerys Graphite & Carbon, Bironico, Switzerland) and poly-tetrafluoroethylene as binder agent (PTFE, Aldrich, St. Louis, MO, USA), with a weight ratio SiOC-DC/CB/PTFE = 70:10:20. After that, few drops of N-methyl-2-pyrrolidinone (Aldrich, St. Louis, MO, USA) were added, and a black slurry was formed. Later on, the working electrodes were prepared via direct deposition over the stainless-steel current collectors, which, during the assembly, were separated by a porous membrane (MF-Milipore mixed cellulose ester). After that, the slurry was dried at 70 ^◦^C for 48 h under vacuum (−0.4 mPa) and then soaked with the electrolyte for at least 48 h. Cyclic voltammetry (CV), electrochemical impedance spectroscopy (EIS), and galvanostatic charge and discharge (GCD) experiments were carried out on a PGSTAT204 potentiostat/galvanostat (Metrohm Autolab, Utrecht, Netherlands) electrochemical analyzer. CV was performed at a potential range from −0.3 to +0.6 V. Different scan rates ranging from 10 to 1000 mVs^−1^ were studied. EIS measurements were analyzed from 0.01 to 105 Hz. GCD experiments were performed employing cut-offs with the same potential window of CV experiments at increasing current densities up to 27 Ag^−1^. The *C_s_* of the samples were calculated from the galvanostatic discharge (GD) curves using (9) [69].

*C_s_* (Fg^−1^) = 4*It_d_*/*m*Δ*V* (9)

where 4 is a coefficient related to full cell configuration (two electrodes), *I* (A) is the current used to discharge the system, *t_d_* (s) is the discharge time, *m* (g) is the mass of both carbon electrodes considering only the active mass material (SiOC-DC), and ΔV (V) is the potential range of the discharge. In order to create the Ragone plots, the *E_d_* of the electrode material was calculated from (10).

*E_d_* (Wh kg^−1^) = *C_s_ V*^2^/2 × 1000 (g/kg) × 1/3600 (Wh J^−1^) (10)

where *C_s_* is the specific capacitance (Fg^−1^), and *V* (V) is the operating voltage (i.e., 0.9 V). The *P_d_* of the electrode was calculated from (11) by dividing the *E_d_* by *t_d_* at certain current densities.

*P_d_* (kW kg^−1^) = *E_d_*/*t_d_* × 3600 (s/h) × 1/1000 (W kW^−1^) (11)

Finally, the cycling stability test at a charge/discharge current density of 0.5 Ag^−1^ of the sample with the highest capacity was determined employing LBT21084 (Arbin Instruments, College Station, TX, USA) testing equipment up to 10,000 cycles.

## 4. Conclusions

Novel hierarchical porous SiOC-DC materials with micro-, meso-, and macropores were obtained for the first time via chlorine etching of SiOC obtained via pyrolysis of sol-gel DMDPS/TREOS hybrids. During the sol-gel process, the high reactivity of Si-H bonds of the TREOS molecules produces a highly cross-linked hybrid and a denser microstructure, which is preserved after pyrolysis in the derived SiOC materials that conditions the microstructure and texture obtained after the subsequent Cl_2_ etching. The C-enriched SiOC material displays 25% carbon, which is deeply enhanced after Cl_2_ etching, whereas the SiOC-DC materials are mainly composed of carbon (≈85%). The SiOC pyrolyzed at 1100 °C presents a dense SiOC network without phase separation, and after Cl_2_ etching, it produces the highest porous microstructure (*S_BET_* = 2499 m^2^ g^−1^) with the largest amount of micropores, derived from the SiC etching, as well as the presence of the largest amount of meso- and macropores, derived from the extraction of bulkier and less-accessible SiO(C) units. The C_free_ phase of this sample (i.e., F11C) has the largest, planar, and least-defective carbon domains (determined using conventional and non-conventional Raman parameters). As the pyrolysis temperature increases, the phase separation into SiO_2_ and SiC starts, and the etching occurs easily without restrictions, giving, as a result, larger pores and thus a decrease in both the *S_BET_* (986–569 m^2^ g^−1^) and pore volume of all types of pores. The C_free_ phase of the latter samples decreases in size and has a very high degree of order and interconnection due to the presence of tortuosity but shows a lower distance between defects than the sample treated at the lowest temperature (i.e., F11C). In F11C, the presence of a hierarchical microstructure with small and big pores can produce the formation of channels that promote ion transportation to the lower-sized pores (i.e., micropores) that are less accessible, enhancing the *C_s_* values. In addition, the presence of larger, less-defective carbon domains without tortuosity seems to facilitate ion transportation and, therefore, the electrochemical response. At low current density, F11C displays a *C_s_* value of 101 Fg^−1^ (0.2 Ag^−1^), which is fairly preserved at a very high current density up to 63 Fg^−1^ (27 Ag^−1^). Relatively high *E_d_* values of 12 and 7 Wh^−1^ kg^−1^ at both low and high *P_d_* values (0.32 and 35 kWkg^−1^) are obtained. Cycling stability studies up to 10,000 cycles were also performed, showing excellent behavior with 98% retention of its initial capacitance.

## Figures and Tables

**Table 1:** Chemical composition of TREOS/DMDPS hybrid.

	C (%)	O (%)	Si (%)	Empirical Formula
F hybrid	27.3	25.7	47.0	SiC_1.35_O_0.96_

**Table 2:** Chemical shift (ppm) and relatively amount (%) of the Si sites extracted from ^29^Si MAS NMR spectra of derived SiOC materials pyrolyzed at different temperatures. n.d. means not determined.

Sample	Q (ppm) (%)	T (ppm) (%)	D (ppm) (%)	X (ppm) (%)
F11	−108.7 (45)	−72.1 (21)	−36.4 (14)	−12.6 (19)
F12	−109.1 (47)	−73.3 (19)	−37.1 (18)	−13.2 (16)
F13	−109.4 (54)	−73.0 (8)	−35.9 (5)	−11.8 (33)
F14	−110.3 (57)	n.d.	n.d.	−15.5 (43)

**Table 3:** Summary of Raman parameters of SiOC and SiOC-DC materials pyrolyzed at different temperatures.

Sample	*D* (cm^−1^)	*W_D_* (cm^−1^)	*G* (cm^−1*)*^	*W_G_* (cm^−1^)	*2D* (cm^−1^)	W_2D_ (cm^−1^)	*L_a_* (nm)	*L_D_* (nm)	*L_eq_* (nm)	Δ*W2D*^−1^ (cm)	*I*_2*D*_ /*I_G_*
F11	1328	141	1560	68	2658	212	5.1	12.2	3.7	0.47	0.24
F12	1341	107	1588	67	2688	146	3.9	10.5	3.1	0.68	0.29
F13	1343	81	1591	63	2683	123	3.9	10.6	4.1	0.81	0.35
F14	1345	71	1591	55	2686	112	3.3	9.7	4.1	0.89	0.39
F11C	1343	96	1589	63	2690	124	5.1	12.1	3.5	0.81	0.27
F12C	1342	62	1580	51	2687	88	3.6	9.1	5.9	1.12	0.57
F13C	1344	59	1585	60	2684	95	3.8	10.6	9.6	1.04	0.78
F14C	1346	52	1582	56	2690	84	3.3	9.7	9.2	1.19	0.86

**Table 4:** C (%) and N_2_ adsorption–desorption data of SiOC and SiOC-DC materials.

	C (%)	*S_BET_* (m^2^ g^−1^)	*V_SP_* (cm^3^ g^−1^)	*V_tot_* (cm^3^ g^−1^)	*V_micro_* (cm^3^ g^−1^)	*V_meso_* (cm^3^ g^−1^)	*V_macro_* (cm^3^ g^−1^)	*D_a_* (nm)
F11	25.4	3		-	-	-	-	12
F12	25.6	6		-	-	-	-	14
F13	24.2	1		-	-	-	-	14
F14	26.5	1		-	-	-	-	16
F11C	84.2	2499	5.95	4.94	0.32	4.13	0.49	10
F12C	86.7	986	2.95	2.12	0.13	1.53	0.47	12
F13C	78.5	724	2.66	2.72	0.17	1.80	0.75	15
F14C	84.3	569	2.46	2.11	0.05	1.60	0.47	17

**Table 5:** Summary of the data (*R_ES_*, *R_CT_*, *f*_0_, and *τ*_0_) obtained from EIS plots of SiOC-DC materials after Cl_2_ etching.

	F11C	F12C	F13C	F14C
*R_ES_* (Ω)	0.4	0.5	0.5	0.6
*R_CT_* (Ω)	9.8	1.7	1.3	1.6
*f*_0_ (Hz)	10.0	39.8	25.0	25.0
*τ*_0_ (s)	0.100	0.025	0.040	0.040

## Data Availability

The data presented in this study are available upon request from the corresponding author.

## Supplementary Materials

The following supporting information can be downloaded at: https://www.mdpi.com/article/10.3390/ijms241813868/s1.
