# Supplementary material for: Highly Porous Carbon Materials Derived from Silicon Oxycarbides and Effect of the Pyrolysis Temperature on Their Electrochemical Response
Source: Int J Mol Sci. 2023 Sep 8;24(18):13868. doi: 10.3390/ijms241813868 (PMC10530778; doi:10.3390/ijms241813868)
Supplement: Supplementary file 1 [file ijms-24-13868-s001.zip › ijms-2580871-supplementary.pdf]

International Journal of *Molecular Sciences*

MDPI

Supplementary information

Highly porous carbon materials derived from silicon oxycarbides and effect of the pyrolysis temperature on their electrochemical response

Jose Merida[1], Maria T. Colomer[2], Fausto Rubio[2] and M. Alejandra Mazo [2,]*

[1] Departamento de Ingeniería Química, Universidad Autónoma de Madrid, C/Tomás y Valiente 7, 28049 Madrid, Spain; josseantoniomeridaa@gmail.com (JM)
[2] Instituto de Cerámica y Vidrio, Consejo Superior de Investigaciones Científicas, C/Kelsen 5, 28049 Madrid, Spain; tcolomer@icv.csic.es (MTC), frubio@icv.csic.es (FR) and sandra@icv.csic.es (MAM)
* Correspondence: sandra@icv.csic.es (MAM)

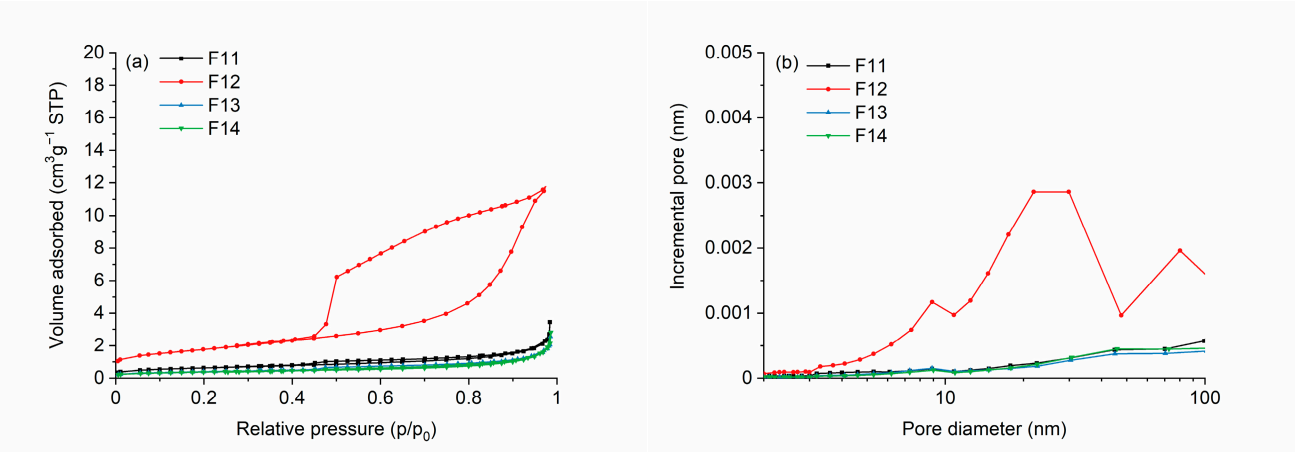

Figure S1. (a) N_2 adsorption-desorption isotherms and (b) pore size distribution of SiOC materials pyrolyzed at different temperatures.

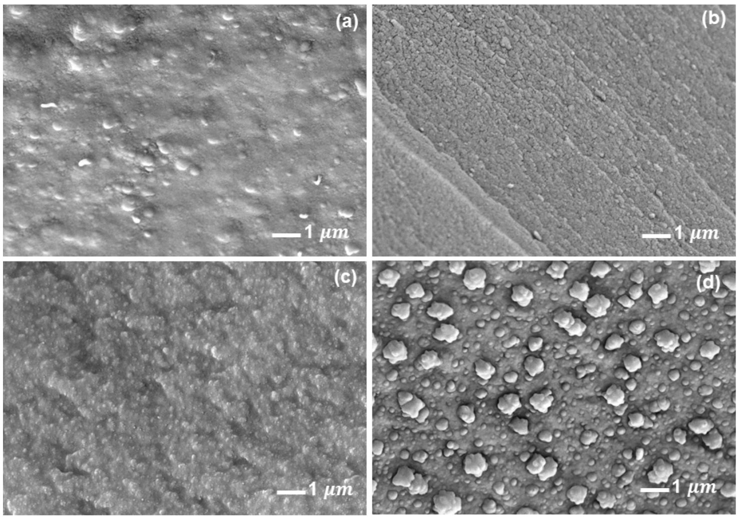

Figure S2. FE-SEM micrographs of SiOC pyrolyzed at different temperatures: (a) 1100 °C, (b) 1200 °C, (c) 1300 °C and (d) 1400 °C.

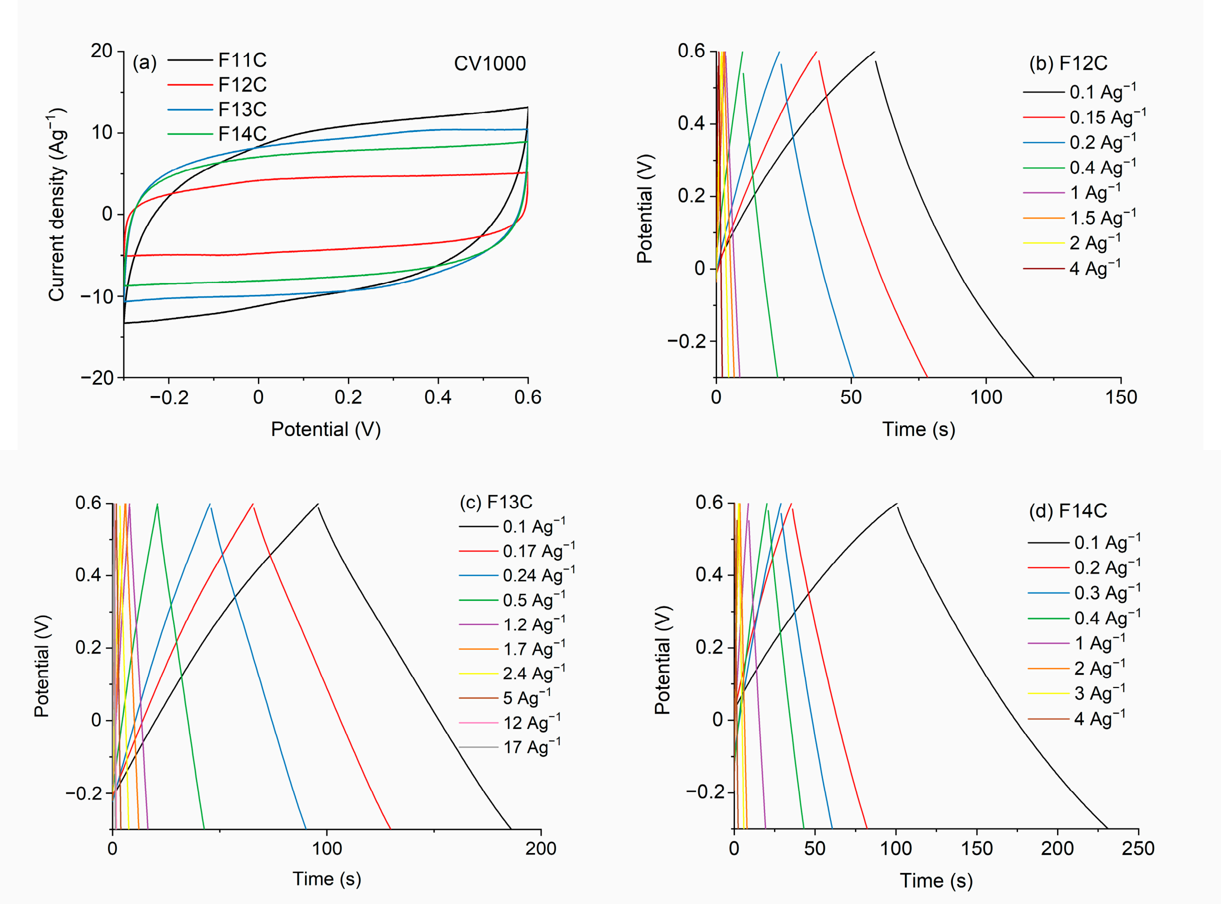

Figure S3. (a) CV curves at scan rate of 1000 $mVs^{-1}$of SiOC-DC materials after Cl_2 etching pyrolyzed at different temperatures, GCD curves at different current densities of (b) F12C, (c) F13C and (d) F14C, respectively.

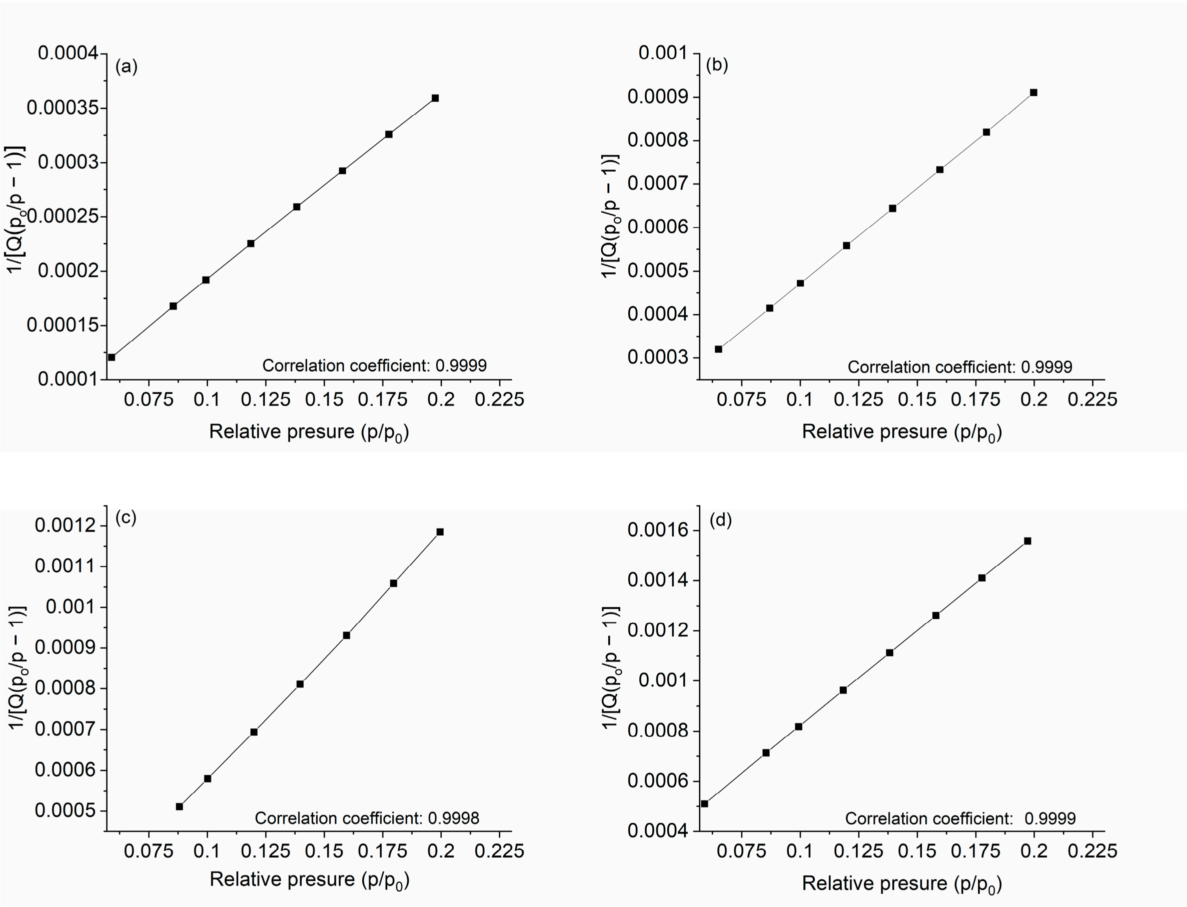

Figure S4. BET plots of SiOC-DC materials after Cl_2 etching pyrolyzed at different temperatures (a) 1100 °C, (b) 1200 °C, (c) 1300 °C and (d) 1400 °C, respectively.
